# Insemination of recipient sows improves the survival to term of vitrified and warmed porcine expanded blastocysts transferred non‐surgically

**DOI:** 10.1111/asj.13453

**Published:** 2020-09-14

**Authors:** Shigeyuki Tajima, Kenzo Uchikura, Takayuki Kurita, Kazuhiro Kikuchi

**Affiliations:** ^1^ Aichi Agricultural Research Center Nagakute Japan; ^2^ Division of Animal Sciences Institute of Agrobiological Sciences National Agriculture and Food Research Organization (NARO) Tsukuba Japan; ^3^ The United Graduate School of Veterinary Science Yamaguchi University Yamaguchi Japan

**Keywords:** artificial insemination, micro volume air cooling, non‐surgical embryo transfer, piglets, vitrified and warmed embryos

## Abstract

This study was performed to evaluate reproductive performance after non‐surgical embryo transfer (Ns‐ET) of 10–15 porcine expanded blastocysts (ExBs) that had been vitrified and warmed (V/W) using the micro volume air cooling (MVAC) method. The effect of asynchrony between the donor and recipient estrous cycle was investigated. Ns‐ET was conducted in recipients whose estrous cycle was asynchronous to that of donors by a delay of 2, 1, or 0 days. In the 2‐day and 1‐day groups, the similar farrowing rates (27.3% and 25.0%) and survival rates to term (13.9% and 15.7%) were obtained after Ns‐ET of V/W ExBs. None of the recipients in 0‐day group farrowed. Artificial insemination (AI) prior to Ns‐ET was then evaluated. Ten–15 V/W ExBs were transferred non‐surgically to 12 recipients whose estrous cycles were asynchronous to that of donors by a 2‐day delay. All of the recipients produced piglets, and all (100.0%) delivered piglets were derived from the transferred V/W ExBs. The survival rate of V/W ExBs to term was 25.2%. These results demonstrate that Ns‐ET of V/W ExBs using MVAC can facilitate piglet production, even if 10–15 embryos are transferred. Moreover, piglets were obtained stably when AI was performed prior to Ns‐ET.

## 1 INTRODUCTION

Embryo transfer (ET) technologies allow cost‐ and labor‐effective transportation of valuable genetic materials and are greatly advantageous for minimizing the risk of disease transmission and avoiding transportation‐related animal stress. Practical application of these techniques is of considerable interest to the pork industry.

Generally, in vivo‐derived porcine embryos can be collected surgically. Although these embryos can be cultured for several days (Blum‐Reckow & Holtz, 1991; Cuello et al., 2016), their long‐term storage by cryopreservation is indispensable for effective application of ET. Recently, there have been significant advances in the cryopreservation of porcine embryos using vitrification (Dobrinsky, Pursel, Long, & Johnson, 2000; Kobayashi, Takei, Kano, Tomita, & Leibo, 1998), and many researchers have reported the production of living piglets derived from vitrified and warmed (V/W) embryos (Beebe, Cameron, Blackshaw, Higgins, & Nottle, 2002; Berthelot, Martinat‐Botté, Locatelli, Perreau, & Terqui, 2000; Berthelot, Martinat‐Botté, Perreau, & Terqui, 2001; Cuello et al., 2016; Fujino et al., 2008; Martinez et al., 2015; Misumi et al., 2013; Misumi, Suzuki, Sato, & Saito, 2003). These reports indicated that, when 20 or more V/W embryos per recipient were transferred surgically, stable pregnancy rates (above 80%) were achieved (Cuello et al., 2016; Fujino et al., 2008; Martinez et al., 2015).

Non‐surgical embryo transfer (Ns‐ET) of V/W embryos, instead of surgical ET (S‐ET), offers considerable promise for improving the efficiency of ET in the pork industry. This technology allows embryos to be deposited deeply in the uterus, and several studies have demonstrated that piglets can be produced from V/W embryos (Cuello et al., 2005; Gomis et al., 2012; Martinez et al., 2015). However, the reproductive performances of recipients after Ns‐ET of V/W embryos was lower than that achieved using surgical procedures (Martinez et al., 2015), and more than 30 V/W embryos were required to achieve an acceptable result (Gomis et al., 2012; Martinez et al., 2015).

On the other hand, the number of in vivo‐derived embryos collected from a single donor per trial was almost limited to 20 (Fujino, Nakamura, Kovayashi, & Kikuchi, 2006). Ideally, therefore, it would be desirable to achieve higher pregnancy and farrowing rates using as small a number of vitrified embryos as possible to make the ET procedure for commercial application (Tajima, Uchikura, Kurita, & Kikuchi, 2020). In this context, Misumi et al. (2013) have succeeded in producing a living piglet after S‐ET of 8–15 V/W expanded blastocysts (ExBs) using the micro volume air cooling (MVAC) method. Omagari et al. (2015) and Sakagami et al. (2016) have also achieved acceptable reproductive performance using this approach. In addition, it is worth noting that the MVAC method employs chemically defined media for vitrification and warming. However, few previous studies have investigated the effectiveness of MVAC for Ns‐ET in terms of reproductive performance.

In our previous study (Tajima et al., 2020), we demonstrated that artificial insemination (AI) prior to S‐ET was effective for assisting the survival to term of a few viable V/W ExBs, and allowed stable production of piglets after surgical transfer of only 10 viable V/W ExBs. Therefore, based on the same rationale, we have hypothesized that AI prior to Ns‐ET might be a similarly effective approach for ensuring successful implantation and maintenance of V/W ExBs after transfer.

In the present study, we examined the efficiency of the MVAC method in terms of reproductive performance after Ns‐ET using fewer than 15 V/W ExBs. We also assessed the potential of AI prior to Ns‐ET for expediting simpler and easier production of piglets from a limited number of transferred V/W ExBs by the MVAC method.

## 2 MATERIALS AND METHODS

### 2.1 Animals

In the present series of experiments, Large White (W), W × Landrace (L) crossbred (WL) and Duroc (D) gilts 8–10 months old were assigned as embryo donors, and W, WL and D sows 13–16 months old were used as embryo recipients. These animals were maintained and fed in accordance with the manuals issued by the Animal Care and Use Committee of Aichi Agricultural Research Center. Briefly, gilts were fed freely until 5 months of age and subsequently restricted to 2.2 kg/head, and water was provided ad libitum. From 5 months of age, they were kept in individual pens throughout the trial. All experiments were performed in accordance with experimental protocols that had been approved by the Animal Care and Use Committee of Aichi Agricultural Research Center.

### 2.2 Chemicals

Chemicals were purchased from Sigma‐Aldrich unless otherwise indicated.

### 2.3 Embryo collection

Embryos were obtained as follows. Detection of estrus in donor gilts was performed twice daily (every 12 hr) by exposure to a mature boar. Donor gilts were given 0.276 mg of sodium cloprostenol (Planate; MSD Animal Health K.K.) intramuscularly twice with a 12‐hr interval during Days 13–16 of the estrous cycle for luteolysis. This was followed by 1,000 IU of equine chorionic gonadotropin (eCG, Peamex; Nippon Zenyaku Kogyo Co., Ltd.,) intramuscularly 24 hr after the second injection of sodium cloprostenol, and then 72 hr later by an intramuscular injection of 500 IU of human chorionic gonadotropin (hCG, Puberogen 1,500 U; Nippon Zenyaku Kogyo) to induce ovulation. The semen used for AI was collected from single L and single D boars in advance on the day of AI or the day before. The samples were examined microscopically to determine sperm characteristics, extended with Modena solution (Weitze, 1991) to 1 × 10^8^ sperms/mL, and stored at 15°C until use. The hormone‐treated gilts were subjected to AI with semen from the same boar two times at 24 hr and 40–42 hr after hCG injection. The semen from the L boar was used for W and WL gilts, and the D boar semen was used to inseminate D gilts. Each time, 50 ml of extended semen (50 × 10^8^ spermatozoa) was introduced via a disposable insemination catheter (No. 0,142,550; Fujihira Industry Co., Ltd.). Embryos were recovered surgically from the donors under general anesthesia 160 hr after hCG injection. A balloon catheter (French size = 16; Nipro Co.) was inserted into the lumen of the uterine horn through a small incision made at a point 5 cm from the uterine body. Each uterine horn was flushed with 50 ml of embryo collection solution, POE‐CM (Mito et al., 2015). The collection solution containing embryos was recovered into a 100‐ml glass tube (Iwaki, 9820TST30NP; AGC Techno Glass), which was then placed in a water bath at 37°C. The embryos were transferred to a 35‐mm plastic dish (Falcon 353,001; Corning) containing 20 mM Hepes‐buffered PBM (Hepes‐PBM) (Mito et al., 2015) and washed twice under a stereomicroscope. After the quality of the embryos had been assessed, their diameters were measured with a micrometer under an inverted microscope at ×200 magnification. Zona‐intact ExBs above 200 μm in diameter were selected and cultured temporarily (within 60 min) in 100 μL PBM using a multidish (Reproplate, IFP9670; Research Institute for Functional Peptides) under a humidified atmosphere of 5% O_2_ and 5% CO_2_ in air at 38.5°C.

### 2.4 Embryo vitrification, warming, and in vitro culture

Vitrification and warming of the embryos were carried out by the MVAC method (Misumi et al., 2013). Media for vitrification and warming were supplied in a commercial kit (PEV‐SK, IFP16PVSK; Research Institute for Functional Peptides). A group of 5–12 ExBs were initially washed twice in Hepes‐PBM, and equilibrated with equilibration solution‐1 (PES‐1; Research Institute for Functional Peptides) followed by equilibration solution‐2 (PES‐2; Research Institute for Functional Peptides) for 5 min each. The equilibrated ExBs were washed briefly in vitrification solution (PVS; Research Institute for Functional Peptides), sequentially transferred into 100‐μL droplets of PVS, and finally ExBs in approximately 1 μL PVS were loaded on the tip of the device designed for MVAC (Embryo‐stick; Misawa Medical Industry). The manipulation of ExBs described above were performed on a warm plate at 38°C. After installation of the ExBs, this device was inserted into a protective sheath and cooled completely in liquid nitrogen (LN_2_) within 1 min of exposure to PVS. The ExBs were stored in LN_2_ for at least 1 month.

Warming and dilution of V/W ExBs were performed as follows. The tip of the device was submerged directly in 3 ml of warming and dilution solution (PWDS; Research Institute for Functional Peptides) which is pre‐warmed for 3 min at 38°C in a 35 mm plastic dish.

The V/W ExBs were then transferred and incubated using a multidish including 100 μL of PBM in a humidified atmosphere of 5% O_2_ and 5% CO_2_ in air at 38.5°C.

### 2.5 Ns‐ET of V/W embryos to recipients

A deep intrauterine catheter (Takumi; Fujihira Industry Co.) (Yoshioka, Noguchi, & Suzuki, 2012) was used for the Ns‐ET of V/W embryos. Estrus synchronization in the recipients was induced in the same manner as that for the embryo donors. Detection of estrus in the recipient sows was performed twice daily (every 12 hr) by exposure to a mature boar. Ns‐ET was conducted without sedation at 120–168 hr after injection of hCG. The V/W ExBs for ET were incubated for 4 hr, washed twice in Hepes‐PBM, placed into a 0.25‐mL plastic straw (NFA121; Fujihira Industry Co.) and maintained at 38.5°C until ET. After thorough cleaning of the perineal area of each recipient, a spiral guide spirette (length: 60 cm) was inserted into the vagina and fixed in the cervix, and then used to guide a deep intrauterine catheter (length: 1.2 m). Prior to insertion, the catheter was rinsed and loaded with 1 ml Hepes‐PBM at 38°C from a 2.5‐mL disposable syringe. The catheter was moved through the cervical canal and propelled forward along one uterine horn until full insertion had been achieved. The 0.25‐mL plastic straw including the ExBs was connected to a 2.5‐mL disposable syringe filled with 1 ml Hepes‐PBM at 38°C using a pipette tip as a connector, and the opposite side of the 0.25‐mL plastic straw was attached to the catheter. The content of the straw was introduced and flushed completely with Hepes‐PBM into the uterus. The period of time from insertion of the spirette to removal of the catheter was recorded. After catheter removal, bends or kinks in the catheter were checked, and if none were evident we assumed that the catheter had been inserted correctly.

The recipients were checked for the return of estrus, and pregnancy was detected using ultrasonography (HS‐1500V; Honda Electronics Co.) on day 30 after estrus or confirmed by spontaneous abortion. Pregnant recipients were allowed to carry pregnancies to term.

### 2.6 Experimental design

#### 2.6.1 Experiment 1: In vitro viability of V/W embryos

The viability of V/W embryos was assessed after in vitro culture for 48 hr. A total of 52 ExBs from five donor gilts were randomly allocated at each collection to either a vitrification group or a control group using fresh embryos. Embryo viability was determined on the basis of full re‐expansion of their spherical shape and development to the hatching or hatched embryo stages.

#### 2.6.2 Experiment 2: Ns‐ET of V/W embryos to asynchronized recipients

A total of 34 sows were used as recipients for Ns‐ET of V/W embryos, and the effects of asynchrony between the donor and recipient estrous cycles on pregnancy, farrowing, and survival rates to term were evaluated. In this experiment, 10–20 vitrified ExBs from one donor were warmed for one trial, and 10–15 V/W ExBs were picked up randomly for transfer. The remaining ExBs were cultured in vitro for 24 hr and their viability was determined as in Experiment 1. A total of 433 ExBs were used for this experiment. The estrous cycle of recipients was controlled by hormone treatment as described above. Ns‐ET was conducted on asynchronous recipients on day 5 (*n* = 11), 6 (*n* = 13) or 7 (*n* = 10) after hCG administration (i.e. the recipient's estrous cycle was asynchronous by a delay of 2, 1, or 0 days relative to that of the donor, and these were designated as the 2‐day, 1‐day, and 0‐day groups respectively).

#### 2.6.3 Experiment 3: Ns‐ET of V/W embryos to recipient gilts after AI

The effects of insemination prior to Ns‐ET of V/W embryos on pregnancy, farrowing, and survival rates to term were evaluated. In this experiment, 12–21 vitrified ExBs from the one donor were warmed for one trial, and 10, 14, or 15 V/W ExBs were picked up randomly for the transfer. The remaining ExBs were cultured in vitro for 24 hr and their viability was determined as in Experiment 1. A total of 192 ExBs were used for this experiment. The estrous cycle of the recipients was controlled in the same manner as that described for Experiment 2.

As an AI/Ns‐ET group, 12 D sows were used. Recipients were subjected to AI with semen from the fixed D or W boar, at a single time point 24 hr after hCG injection. Subsequently, Ns‐ET was conducted at 5 days after hCG administration, and the predicted estrous cycle of the recipients was delayed by 2 days compared with the donors. Eight recipients that underwent AI from the D boar received V/W ExBs that were not derived from D, and four recipients that underwent AI from the W boar received V/W ExBs derived from D. These procedures allowed the origin of the piglets to be distinguished on the basis of coat color (i.e., piglets derived from AI with D semen were wholly brown, whereas those from V/W ExBs were white or spotted). In addition, nine sows were subjected to AI using the same boars (five sows with the D boar and the remains four with W boar) in the same way as for the AI/Ns‐ET group, but ET was not performed (designated the AI group).

### 2.7 Statistical analysis

Data for survival rates to term and in vitro viability of V/W ExBs were analyzed by ANOVA using the General Linear Models procedures of the Statistical Analysis System (Ver. 9.2; SAS Institute Inc.). Other data from the ET experiment were analyzed by Fisher's exact test. Percentage data were arcsine‐transformed before the analysis. *p*‐values of <.05 were considered to indicate significance.

## 3 RESULTS

### 3.1 Experiment 1

As shown in Table 1, no significant difference in in vitro viability was observed among the V/W embryo and control groups (90.0 ± 4.2% and 100%, respectively). The hatching and hatched rate of V/W ExBs after 48 hr of incubation was significantly lower (*p* < .05) than that in the control group (66.7 ± 3.7% and 90.9 ± 5.7% respectively).

**Table 1:** In vitro viability of VW porcine expanded blastocysts by MVAC method

Source of embryos	No. of embryos examined	No. (%)^a^ of viable embryos after culture for 48 hr		
		Total	Re‐expanded	Hatching/hatched
Control	22	22 (100)	2 (9.1 ± 4.1)	20 (90.9 ± 5.7)^b^
VW	30	27 (90.0 ± 4.2)	7 (23.3 ± 4.2)	20 (66.7 ± 3.7)^c^

Note

Five replicated trials were performed for each embryo source.

Abbreviations: MVAC: micro volume air cooling; VW, vitrified and warmed.

^a^ Means ± *SEM*.

^b,c^ Within each column, values with different subscripts differ significantly (*p* < .05).

### 3.2 Experiment 2

In this experiment, a total of 433 ExBs were used, where 355 and 78 were subjected to Ns‐ET and in vitro culture respectively. Four of 34 recipients were excluded due to incorrect insertion, which was predicted by the presence of bends or kinks in the catheter after removal. In this experiment, a total of 30 recipients were analyzed. None of those recipients showed symptoms of uterine infection such as vaginal discharge after Ns‐ET.

As shown in Table 2, no recipient became pregnant in the 0‐day group. However, three recipients each in the 2‐day and 1‐day groups become pregnant and farrowed live piglets, with farrowing rates of 27.3% and 25.0% respectively. The survival rates to term of the transferred V/W ExBs in the 2‐day and 1‐day groups were 13.9% and 15.7% respectively. There were no significant differences in the pregnancy rate, farrowing rate, or survival rate to term of transferred V/W ExBs between the 2‐day and 1‐day groups. The mean (±*SE*) birth weight of piglets in the 2‐day and 1‐day groups were 1.47 ± 0.10 kg and 1.39 ± 0.07 kg (data not shown in Table 2), with no significant inter‐group difference. In addition, the in vitro viability of V/W ExBs after culture for 24 hr did not differ among the three groups.

**Table 2:** Effect of asynchrony between donor and recipient estrous cycle on reproductive performance after non‐surgical transfer (Ns‐ET) of 10 to 15 vitrified and warmed embryo, and in vitro viability of vitrified and warmed embryos by MVAC method

asynchrony between donors and recipients (days)^a^	Ns‐ET							In vitro culture	
	Total number of recipients	Embryos transferred (per recipient)	Time required for Ns‐ET (min)	No. of pregnant recipients (%)	No. of recipients farrowed (%)	No. of piglets	Survival rate to term of transferred embryos^b^ (%)	No. of embryos	Viability^c^ after 24 hr (%)
2‐day	11	137 (12.5 ± 0.7）	6.0 ± 0.4	3 (27.3)	3 (27.3)	3,8,8	13.9 ± 6.7	38	30 (78.9 ± 6.1)^d^
1‐day	12	140 (11.7 ± 0.4）	6.2 ± 0.4	3 (25.0)	3 (25.0)	4,8,10	15.7 ± 8.8	22	17 (77.3 ± 9.0)^e^
0‐day	7	78 (11.1 ± 0.5）	6.8 ± 0.5	0	0	*n*.d	0.0	18	13 (72.2 ± 9.5)^f^

Note

Means ± *SEM*.

Abbreviation: MVAC: micro volume air cooling.

^a^ Estrus cycle in recipients were asynchronous delay.

^b^ Calculated as follows: (number of piglets/number of transferred embryos) × 100.

^c^ Blastocysts showing re‐expansion or development to the hatching/hatched blastocyst stages.

^d^ Nine replicated trials were performed for each embryo source.

^e^ Eleven replicated trials were performed for each embryo source.

^f^ Seven replicated trials were performed for each embryo source.

### 3.3 Experiment 3

None of 12 recipients showed symptoms of uterine infection such as vaginal discharge after Ns‐ET. As shown in Table S1, all recipients became pregnant and farrowed 126 piglets in total (10.5 ± 0.8/L). A total of 86 piglets from 12 recipients were found to be derived from embryos after fertilization by AI on the basis of piglet coat color, whereas 40 piglets from 12 recipients were generated from V/W and transferred ExBs. As shown in Table 3, the survival rates to term of V/W ExBs were not different (26.9 ± 5.5% and 20.0 ± 5.8%) when inseminated with semen from D and W boars respectively. The farrowing rate when derived from Ns‐ET was 100%. The birth weight of piglets originating and not originating from transferred embryos was 1.45 ± 0.05 kg and 1.40 ± 0.03 kg, respectively, with no significant difference between piglet origins (data not shown in Table 3). In addition, the in vitro viability of V/W embryos after 24 hr of culture was 81.8% in both groups (Table 3). In the AI group, all five sows inseminated with D semen farrowed and produced 50 piglets in total (10.0 ± 0.5/litter), and all four sows inseminated with W semen farrowed and produced 26 piglets in total (6.5 ± 1.8/litter), with no difference between semen origins (Table 3). The birth weights in the two groups were not different (1.33 ± 0.04 kg and 1.35 ± 0.06 kg respectively) (data not shown in Table 3).

**Table 3:** Results of non‐surgical embryo transfer (Ns‐ET) after artificial insemination (AI) or only AI, and in vitro viability of vitrified and warmed embryos by MVAC method

Exp. group	Ns‐ET								In vitro culture	
	Total number of recipients	Time required for ET (min)	No. of pregnant recipients (%)	No. of recipients farrowed (%)	Total No. of piglets (Means ± *SEM*）	No. of recipients producing piglets derived from Ns‐ET (%)	No. piglets derived from Ns‐ET (%）	Survival rate to term of transferred embryos (%)^a^	No. of embryos	Viability^b^ after 24 hr (%)
AI/Ns‐ET^c^	8	5.9 ± 0.2	8 (100)	8 (100)	92 (11.5 ± 0.5)	8 (100)	32 (4.0 ± 0.8)	26.9 ± 5.5	22	18 (81.8 ± 7.4)^e^
AI^c^	5	*n*.d	4 (100)	4 (100)	50 (10.0 ± 0.5)	*n*.d	*n*.d	*n*.d	*n*.d	*n*.d
AI/Ns‐ET^d^	4	5.9 ± 0.2	4 (100)	4 (100)	34 (8.5 ± 2.0)	4 (100)	8 (2.0 ± 0.6)	20.0 ± 5.8	11	9 (81.8 ± 12.5)^f^
AI^d^	4	*n*.d	4 (100)	4 (100）	26 (6.5 ± 1.8)	*n*.d	*n*.d	*n*.d	*n*.d	*n*.d

Note

Means ± *SEM*. Detailed data for AI/Ns‐ET are available in Table S1.

Abbreviation: MVAC: micro volume air cooling.

^a^ Calculated as follows: (number of piglets/number of transferred embryos) × 100.

^b^ Blastocysts showing re‐expansion or development to the hatching/hatched blastocyst stages.

^c^ Inseminated with fixed Duroc semen.

^d^ Inseminated with fixed Large White semen.

^e^ Five replicated trials were performed for each embryo source.

^f^ Four replicated trials were performed for each embryo source.

## 4 DISCUSSION

The present study confirmed that non‐surgical deep intrauterine transfer of V/W ExBs by the MVAC method facilitated piglet production, even though the number of transferred embryos was less than 15 (range, 10–15). Moreover, when combined with AI prior to Ns‐ET, normal live piglets were obtained effectively and stably.

Martinez et al. (2015) suggested that 30 V/W embryos would be adequate for surgical transfer, implying that more V/W embryos would be needed for Ns‐ET than for S‐ET. Although Gomis et al. (2012) and Martinez et al. (2015) achieved acceptable reproductive performance by Ns‐ET using 40 V/W embryos, only 20 in vivo‐derived embryos could be collected from a donor per single trial (Fujino et al., 2006), and these statuses would make it difficult to accumulate a sufficient number of embryos for distribution to a number of recipients. Ideally, it would be desirable to achieve efficient pregnancy and farrowing rates using as few cryopreserved embryos as possible. In this context, it is considered that the combination of AI and Ns‐ET for V/W ExBs by the MVAC method is a valuable procedure for practical application.

The present Experiment 1 was able to confirm the viability of V/W ExB using MVAC. The viable and hatching rates of V/W ExBs after 48 hr of in vitro culture (Table 1) were similar to those reported previously (Misumi et al., 2013; Sakagami et al., 2016). Here, using these V/W ExBs, we confirmed that it was possible to conduct subsequent embryo transfer experiments.

In Experiment 2, Ns‐ET of 10–15 V/W ExBs was conducted using MVAC. Recipients in the 0‐day group failed to become pregnant. On the other hand, recipients in the 2‐day and 1‐day groups showed both pregnancy and fallowing rates of 27.3% (3/11) and 25.0% (3/12), respectively, and no difference was observed (Table 2). One of the major factors responsible for the success of ET is the degree of estrous synchrony between recipient and donor (Angel et al., 2014). It has been hypothesized that embryo manipulation and/or in vitro culture might cause a transitory delay of porcine embryo development (Almiñana et al., 2010; Macháty, Day, & Prather, 1998) and that porcine embryos might show tolerance to a “less advanced” uterine environment (Angel et al., 2014). When in vivo‐ or in vitro‐produced fresh (non‐vitrified) blastocysts are deposited into the uterine horn using non‐surgical procedures, indices of reproductive performance such as pregnancy, farrowing or efficiency of piglet production may be enhanced in recipients whose estrous cycles are asynchronous with a 1‐ or 2‐day delay relative to the donors (Angel et al., 2014; Yoshioka et al., 2012). In the present study also (Table 1), the development of V/W ExBs was generally delayed in comparison with fresh ExBs. Thus, appropriate conditions for development of transferred V/W ExBs might also allow adaptation to a less advanced uterine environment. In previous studies (Cuello et al., 2005; Gomis et al., 2012; Martinez et al., 2015) of Ns‐ET for V/W blastocysts, asynchronous recipients whose estrous cycles were delayed by 1 day relative to the donors produced living piglets. In addition, piglet production from S‐ET of V/W blastocysts by the MVAC method was demonstrated for recipients whose estrous cycles were asynchronous with those of the donors by a 1‐ or 2‐day delay (Misumi et al., 2013; Omagari et al., 2015; Sakagami et al., 2016). Our results are similar to those reports; therefore, recipients whose estrous cycle are asynchronous with a 1‐ or 2‐day delay are adequate for non‐surgical deep intrauterine transfer of V/W ExBs using the MVAC method. In Experiment 3, we were concerned that development of transferred V/W ExBs might be delayed and that embryos derived from insemination might hinder implantation. For these reasons, it may be necessary to transfer V/W ExBs to recipients whose estrous cycles are asynchronous with some degree of delay. Thus, it was expected that Ns‐ET for recipients with an asynchronous estrous cycle 2 days behind that of donors would allow the transferred V/W ExBs to develop effectively for improved implantation. We observed that 12 recipients produced piglets derived from both transferred ExBs and AI, thus confirming that recipients with a 2‐day delay in their estrous cycle relative to the donors were appropriate for this procedure. However, as we did not compare other conditions of synchrony (1‐day delay), it will be necessary to perform additional experiments to derive more appropriate conditions of synchrony and transfer for V/W ExB development.

When the average number of surgically transferred V/W ExBs using the MVAC method is less than 15, the farrowing rate and survival rate to term are more than 70% and 20% respectively (Omagari et al., 2015). However, in the present study, the farrowing rate and survival rate to term after Ns‐ET were markedly inferior to those by S‐ET. Martinez et al. (2015) compared the effectiveness of surgical with non‐surgical transfer of 30 V/W embryos and demonstrated that the farrowing rate and survival rate to term by Ns‐ET were significantly lower. Furthermore, similar results were obtained using both fresh in vivo‐derived and in vitro‐produced embryos (Hazeleger, Bouwman, Noordhuizen, & Kemp, 2000; Martinez et al., 2004; Yoshioka et al., 2012) among both S‐ET and Ns‐ET. Although the reason for the disadvantageous outcome of Ns‐ET has not been clarified, it is probable that a larger number of transferred embryos may remain viable just after transfer or for a certain period after, but are lost from the uterine cavity or lose their survival ability to term before/after implantation. Martinez et al. (2015) demonstrated that transfer of a higher number of embryos overcame the negative effects of Ns‐ET. On the other hand, we have applied AI prior to ET to achieve acceptable reproductive performance by S‐ET (Tajima et al., 2020). In that previous study, we demonstrated that AI prior to S‐ET was effective for assisting development to term even when a limited number (10) of V/W ExB were transferred. It is well‐known that at least four conceptuses are needed (i.e., that four embryos need to be implanted) for establishment and maintenance of pregnancy during early gestation in pigs (Polge, Rowson, & Chang, 1966). If less than four viable embryos implanted during early gestation, they could not complete fetal development and resulted in failure of pregnancy in many cases. To ensure pregnancy in the recipients, we considered that creation of adequate “numerical support” from embryos obtained by AI prior to ET might be promising, and consequently it was expected that even if the number of V/W ExBs transferred was limited, a few of them would develop to term. It seems that when they are used also for Ns‐ET, similar effects might be expected. As shown in Table 3, when 12 recipient sows were inseminated prior to Ns‐ET of 10–15 V/W ExBs, all of the recipients farrowed and all (100%) of the sows produced piglets derived from V/W ExB. The survival rate to term of V/W ExB after Ns‐ET without AI was 25.2%, regardless of the semen origin (Table S1). These results were equivalent to the reproductive performance of S‐ET using MVAC method reported previously (Misumi et al., 2013; Omagari et al., 2015; Sakagami et al., 2016). Consequently, it is considered that AI prior to Ns‐ET might overcome the disadvantage of Ns‐ET.

In the present Ns‐ET series, we experienced no difficulty with catheter insertion into the uterine horn in Experiment 2 except in four recipient sows, which were excluded from the study for this reason. As three of these four cases were in the 0‐day group, it is suggested that 0‐day is not an adequate time point for Ns‐ET. However, we will need to perform further experiments to investigate this issue further. The time period from insertion of the spirette to removal of the catheter was less than 7 min. The tip of the catheter was inserted almost 60 cm anterior to the tip of the guide spirette, and we recognized no kinks or bends in the catheter after removal except in the four recipients mentioned above. It is inferred that V/W ExBs were deposited at least in the mid to caudal quarter of the uterine hone (Yoshioka et al., 2012). In addition, none of the recipients showed signs of uterine infection such as vaginal discharge after Ns‐ET. It is therefore considered that the procedure we used in this study is safe and well tolerated for recipient sows.

In conclusion, the results of the present study demonstrate that non‐surgical deep intrauterine transfer of V/W ExBs by the MVAC method facilitates piglet production, even with fewer than 15 transferred embryos. Although the reproductive performance of Ns‐ET is inferior to that of S‐ET, AI prior to ET can overcome this disadvantage. When is paired with AI prior to non‐surgical transfer of 10–15 V/W ExBs by the MVAC method, normal live piglets derived from ET can be obtained effectively and stably.

## CONFLICT OF INTEREST

All authors declare no conflict of interests.

## Supporting information

Table S1
