# Supplementary material for: Insemination of recipient sows improves the survival to term of vitrified and warmed porcine expanded blastocysts transferred non‐surgically
Source: Anim Sci J. 2020 Sep 14;91(1):e13453. doi: 10.1111/asj.13453 (PMC7539913; doi:10.1111/asj.13453)
Supplement: Supplementary file 1 — Table S1 [file ASJ-91-e13453-s001.docx]

**Supplemental Table 1.** Non-surgical embryo transfer (Ns-ET) of 10 to15 vitrified/warmed embryos after artificial insemination (AI) and piglet production by MVAC method

| Trial No. | No. of embryos | semen for AI | Pregnancy | No. of piglets | No. of piglets derived from Ns-ET | Survival rate to term of transferred embryos (%)^a^ |
| --- | --- | --- | --- | --- | --- | --- |
| 1 | 15 | D | + | 11 | 2 | 13.3 |
| 2 | 15 | D | + | 12 | 2 | 13.3 |
| 3 | 14 | D | + | 13 | 2 | 14.3 |
| 4 | 15 | D | + | 13 | 3 | 20.0 |
| 5 | 15 | D | + | 10 | 4 | 26.7 |
| 6 | 15 | D | + | 13 | 4 | 26.7 |
| 7 | 15 | D | + | 10 | 7 | 46.7 |
| 8 | 15 | D | + | 10 | 8 | 53.3 |
| Subtotal | 119 |  |  | 92 | 32 | 26.9 ± 5.5 |
| 9 | 10 | W | + | 9 | 1 | 10.0 |
| 10 | 10 | W | + | 14 | 1 | 10.0 |
| 11 | 10 | W | + | 5 | 3 | 30.0 |
| 12 | 10 | W | + | 6 | 3 | 30.0 |
| Subtotal | 40 |  |  | 34 | 8 | 20.0 ± 5.8 |
| Total | 159 |  |  | 126 | 40 | 25.2± 4.1 |

Mean ± SEM

^a^ Calculated as follows: (number of piglets/number of transferred embryos) × 100

MVAC: micro volume air cooling
